# A 2024 Update on Menin Inhibitors. A New Class of Target Agents against KMT2A-Rearranged and NPM1-Mutated Acute Myeloid Leukemia

**DOI:** 10.3390/hematolrep16020024

**Published:** 2024-04-18

**Authors:** Anna Candoni, Gabriele Coppola

**Affiliations:** Section of Haematology, Department of Medical and Surgical Sciences, University of Modena and Reggio Emilia, Via del Pozzo 71, 41123 Modena, Italy; gabriele.coppola@studenti.unina.it

**Keywords:** menin, menin inhibitors, acute leukemia, KMT2A, NPM1, HOX genes, revumenib, ziftomenib

## Abstract

Menin inhibitors are new and promising agents currently in clinical development that target the HOX/MEIS1 transcriptional program which is critical for leukemogenesis in histone-lysine N-methyltransferase 2A-rearranged (KMT2Ar) and in NPM1-mutated (NPM1mut) acute leukemias. The mechanism of action of this new class of agents is based on the disruption of the menin–KMT2A complex (consisting of chromatin remodeling proteins), leading to the differentiation and apoptosis of AML cells expressing KMT2A or with mutated NPM1. To date, this new class of drugs has been tested in phase I and II clinical trials, both alone and in combination with synergistic drugs showing promising results in terms of response rates and safety in heavily pre-treated acute leukemia patients. In this brief review, we summarize the key findings on menin inhibitors, focusing on the mechanism of action and preliminary clinical data on the treatment of acute myeloid leukemia with this promising new class of agents, particularly revumenib and ziftomenib.

## 1. Introduction

Acute myeloid leukemia (AML) is a genetically heterogeneous group of myeloid neoplasms that result from the uncontrolled proliferation of clonal hematopoietic cells, leading to ineffective hematopoiesis and life-threatening cytopenias. AML accounts for 80% of acute leukemias in adults with a median age at diagnosis of 68 years [1,2]. Over the last decade, a better understanding of the pathophysiology and genomic characterization of AML has improved our knowledge on the prognostic and therapeutic landscape of this neoplastic disease [1,2,3]. However, although the prognosis of AML has improved over the last decade, several challenges remain, particularly in relapsed or refractory AML, in elderly patients, and in AML with high cytogenetic-molecular risk [1,2,3]. In this scenario of rapid and interesting changes, menin inhibitors represent a novel class of drugs that have shown promising results in histone-lysine N-methyltransferase 2A (KMT2A)-rearranged (KMT2Ar) and in NPM1-mutated (NPM1mut) acute leukemias [1,2]. In this brief review, we outline the key findings on menin inhibitors, focusing on the mechanism of action and on preliminary clinical applications in AML of this emerging class of agents.

## 2. Role of Menin in KMT2Ar and NPM1mut AML

**Figure 1:**
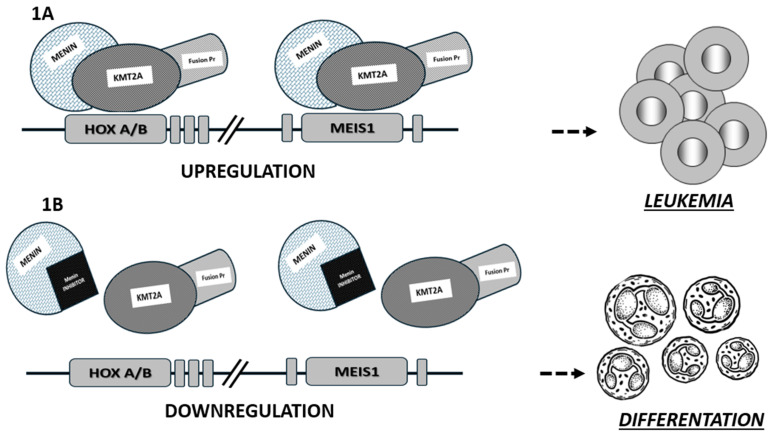
Cofactor menin is necessary for KMT2A to bind HOX gene promoters. In detail, KMT2A binding menin acts as a transcriptional upregulator of HOX genes and their cofactor MEIS1 (**A**). Disruption of this chromatin complex (by binding of the menin inhibitors and menin protein) leads to inhibition of the leukemogenic transcription program (downregulation of HOX genes) without affecting normal hematopoiesis (**B**).

Menin is a nuclear scaffold protein involved in many biological processes (including the regulation of hematopoiesis and myeloid proliferation) and is encoded by the MEN1 gene (containing 10 exons and located on chromosome 11q13), which is mutated in patients with multiple endocrine neoplasia type 1 (MEN1). Menin has a tumor suppressor function in endocrine glands, and in this setting, menin inactivation (lack of enzymatic or DNA-binding activity) represents an oncogenic transformation. However, menin can also have oncogenic properties in various tissues and has numerous binding partners, some of which are still unknown [4,5]. It interacts with DNA through its nuclear localization sequences located in the C-terminal region, resulting in the regulation of gene expression [6,7]. This protein acts as a link between transcription factors and epigenetic effectors. A dependence on menin is known in KMT2Ar leukemias, in NPM1mut AML and in other types of neoplasms. In this context, the interactions of menin with the KTM2A protein are considered critical for the leukemic transcriptional program in AML through the upregulation of HOX/MEIS1 gene expression (see Figure 1) [8,9,10,11].

The KMT2A proto-oncogene (formerly known as the mixed-lineage leukemia-MLL gene) is located on chromosome 11q23 and encodes for a transcription factor (KMT2A) involved in embryonic development and hematopoiesis (as an important epigenetic regulator). KMT2A regulates key genes such as HOXA and HOXB, MEIS1, PBX3, MEF2C and CDK6. Furthermore, in NPM1mut AML, KMT2A regulates the oncogenic expression of HOXA, MEIS1 and FLT3, thereby stimulating the proliferation of myeloid progenitor cells [6,7]. KMT2A rearrangements are not only restricted to the myeloid leukemia, but also occur in acute lymphoblastic leukemia (ALL) and in mixed-phenotype acute leukemia (MPAL). Rearrangements in KMT2A are present in approximately 20% of children with de novo AML and in 5–10% of adults and are associated with a poor prognosis with higher rates of relapse and resistance to chemotherapy [6,7,12].

The KMT2A has more than 80 fusion partners, mostly transcriptional cofactor proteins such as AF4 (~36%), AF9 (~19%), ENL (~13%), AF10 (~8%), ELL (~4%) and AF6. In KMT2Ar AML, all of these fusion proteins interact with chromatin-associated protein complexes, including menin. These interactions, through epigenetic modulation of transcription, upregulate genes critical for hematopoietic cell proliferation and differentiation, including homebox (HOX A/B) and MEIS1 (myeloid ecotropic virus insertion site 1 genes). Expression of HOX A/B and MEIS1 genes is high in the immature stem cell population and decreases during maturation and differentiation of the hematopoiesis [7,13]. KMT2A is a crucial regulator of HOX genes transcription which affect the proliferation and differentiation of myeloid progenitors. The role of HOX gene deregulation in the setting of leukemogenesis has been verified in both in vitro and in vivo models, highlighting the role of KMT2A fusion proteins in the aberrant stimulation of proliferation and blockade of hematopoietic differentiation [6,9,10]. 

In KMT2Ar leukemia, the interaction of KTM2A fusion proteins with menin is a key driver of leukemogenesis, as menin is an essential cofactor for binding to HOX gene promoters. Menin binds to the N-terminal region of KMT2A (a highly conserved region in all KMT2A fusion proteins), and upon menin binding, KMT2A fusion proteins translocate to the nucleus.

Nuclear localization leads to the stimulation of aberrant transcription of HOXA and other genes, which is critical for KMT2Ar AML pathogenesis [8]. The fundamental role of the interaction between menin and the N-terminal region of KMT2A in leukemogenesis has been demonstrated in many in vivo and in vitro studies, where loss of menin binding eliminates the oncogenic properties of KMT2A fusion proteins (see Figure 1) [9,10].

Nucleophosmin 1 (NPM1) is one of the most common gene mutations in AML, found in one-third of newly diagnosed AMLs occurring in 25–30% of adult AML patients and in 10% of pediatric AML patients. The NPM1-mutated AML is classified as a distinct entity in the 2022 World Health Organization (WHO 2022) and European Leukemia Network (ELN 2022) classifications of myeloid neoplasms [1,2,3].

NPM1 is a chaperone protein, with multiple biological functions, that shuttles between the nucleus and the cytoplasm. The N-terminal portion of NPM1 contains two leucine-rich nuclear export signals (NESs), while the central acidic core contains a bipartite nuclear localization signal and the C-terminal portion contains a nucleolar localization signal (NoLS), which is responsible for the primary nucleolar localization of wild-type NPM1 (NPM1wt) [14]. Under physiological conditions, NPM1wt is essential in different phases of the cell cycle, participates in ribosome biogenesis (transport of ribosomal components) and cell growth, prevents uncontrolled proliferation, interacts with p53 in the event of oxidative damage and plays an important role in the prevention of mutagenesis (through the regulation of apoptosis and DNA repair) [15,16]. Insertions in exon 12 of the NPM1 gene (type A and B) are the most common mutation subtypes. The mutated NPM1 has a stable cytoplasmic delocalization. Given the pleomorphic function of NPM1, this is a critical step in leukemogenesis. The NPM1-mutated AML has a gene expression profile similar to KMT2Ar leukemias with the upregulation of HOX and MEIS1 genes [14,17]. Indeed, gene editing studies have confirmed the dependence of NPM1mut on menin and MEIS1 to activate the leukemic transcriptional program, although the exact mechanism by which NPM1 induces the overexpression of HOX, MEIS1 and other genes is still less clear [14,17]. In addition, some other leukemias overexpressing HOX and MEIS1 genes might respond to menin inhibition such as leukemias with rearrangement of the nucleoporin 98 gene (NUP98) [18]. 

## 3. Targeting Menin in AML

A better understanding of the molecular landscape and pathophysiological pathways of hematopoietic proliferation has led to the development of therapeutic strategies beyond standard chemotherapy [1,2,3,19]. Therefore, the knowledge of the pathological interactions between menin and KMT2A in KMT2Ar leukemias and NPM1mut AML, as well as the underlying mechanism of action analyzed above, has led to the recognition of a new promising target for therapies in this subset of leukemias. Architectural analysis of menin shows that its structure is highly conserved. Menin has a central cavity that is the binding site for protein–protein interactions. After a detailed architectural characterization of the protein, it became clear that the central pocket of menin is a very interesting site for the possible inhibition of interaction with KMT2A, as shown in Figure 1 [10].

In the menin–KMT2A interaction, two short motifs in the N-terminal fragment of KMT2A contribute to menin binding, called menin binding motif 1 (MBM1) and menin binding motif 2 (MBM2). MBM1 has a higher affinity for menin than MBM2 and its binding site to menin is better characterized. However, MBM1 and MBM2 both compete for binding to the proximal sites of menin. Some studies have shown that targeting MBM1–menin binding could lead to efficient disruption of the menin–KMT2A interaction. Therefore, the MBM1–menin interaction became the most promising site for the development of menin inhibitors based on a peptide corresponding to the MBM1 fragment [9,20].

In brief, as shown in Figure 1, the mechanism of action of menin inhibitors is based on the disruption of menin–KMT2A binding, preventing the formation of the specific fusion complex on chromatin, leading to the downregulation of HOX and MEIS1 transcription and inhibition of leukemogenesis.

One potential biomarker for monitoring the response to menin inhibitors is the evaluation of HOX (A and/or B) gene expression together with levels of its cofactor, MEIS1. However, in the absence of a validated assay to assess the expression of these genes, a possible surrogate marker could be the analysis of leukemia genotypes known to have this aberrant gene expression signature. This could also lead to the identification of leukemias with different genotypes other than NPM1mut or KMT2Ar that might respond to these new drugs. However, future studies are needed to identify a potential biomarker for response to menin inhibition [5].

In addition, in some patients on menin inhibitor therapy, one or more mutations in the MEN1 gene were detected prior to overt morphologic relapse, representing a mechanism of acquired resistance during therapy. These findings suggest the importance of monitoring patients for the emergence of MEN1 mutations during menin inhibitor treatment to predict morphologic relapse and allow timely evaluation of other therapeutic strategies [21].

## 4. Menin Inhibitors: Ongoing Clinical Trials in AML

Several early-phase clinical trials are investigating the safety and efficacy of menin inhibitors in AML with KMT2Ar or NPM1mut AML. Very encouraging safety and efficacy data are currently available from studies of two menin inhibitors: SNDX-5613 (revumenib) and KO-539 (ziftomenib) [22,23,24,25,26,27,28,29]. Ongoing phase I/II clinical trials evaluating menin inhibitor therapy are listed in Table 1.

### 4.1. Revumenib

Revumenib (formerly SNDX-5613) is a potent, oral menin inhibitor that selectively blocks interactions between KMT2A and menin, thereby silencing leukemic transcriptional programs. Revumenib appears to be effective in reducing the overexpression of the MEIS1 and HOX genes, critical leukemogenic targets analyzed above.

The **AUGMENT-101** trial (**NCT04065399**) is a first-in-human phase (Ph) 1 study to determine the safety, maximum tolerated dose (MTD), recommended phase 2 dose (RP2D) and pharmacokinetic (PK) profile of revumenib in patients with relapsed or refractory (R/R) acute leukemia [22,23,24,25]. Patients enrolled in this trial are heavily pretreated and have a very poor prognosis. Revumenib is a substrate of CYP3A4, and therefore, the study included two cohorts: Arm A included patients who received strong CYP3A4 inhibitors, while Arm B included patients who did not receive strong CYP3A4 inhibitors. The two arms also differ in the dose of revumenib. The initial results of this study were reported in November 2022 on 68 patients with R/R acute leukemia (including 60 patients with KMT2Ar or NPM1mut) with a median age of 43 years (range, 1–79) and 51 years in adults (range 19–79). The most common adverse events (AEs) were asymptomatic grade 3 QTc prolongation without ventricular arrhythmia and grade 2 differentiation syndrome (DS), successfully treated with steroids and hydroxyurea. Other grade ≥3 treatment-related adverse events (TRAEs) reported in this study were diarrhea (3%), fatigue (3%), anemia (3%), tumor lysis syndrome (2%), neutropenia (2%), thrombocytopenia (2%), hypercalcemia (2%) and hypokalemia (2%), but no treatment discontinuations or deaths due to TRAEs were reported. The overall response rate (ORR) was 53% (32/60); complete remissions (CRs), CRs with incomplete hematologic recovery (CRh) and CRs with incomplete platelet recovery (CRp) were reported in 38% of patients (23/60). In addition, patients with KMT2Ar or NPM1mut leukemia had an ORR of 59% (27/46 pts) and 36% (5/14 pts), respectively. Of the 32 responders, 18 achieved MRD negativity (56%) and 12 (38%) underwent allogeneic stem cell transplantation (HSCT). Eight patients did not respond to revumenib, but all had wild-type KMT2A and wtNPM1 or genotypes that are not susceptible to menin inhibition.

Median follow-up in the responder population was 4.6 months (range 0.3–21.9); median duration of response (DOR) was 9.1 months (95% CI: 2.7-NR), and median overall survival (OS) was 7 months (95% CI: 4.3–11.6; range 0.3–21.9).

These preliminary data in R/R leukemia are impressive and suggest that menin inhibition with revumenib is a successful therapeutic strategy in acute leukemia with KMT2Ar or NPM1mut. Treatment was associated with a low incidence of ≥grade 3 TRAEs and with a consistent ORR in patients refractory to multiple prior lines of therapy [22,25].

An update of the phase 1 study was recently reported with safety data from 131 patients and efficacy data in 80 patients with R/R KMT2Ar leukemia. In 51 adults with AML, the CR+CRh was 28% and the ORR was 59%. Responses in ALL or other leukemia subtypes were as consistent as in AML, with 27% CR+CRh and 53% ORR. In the safety population, 24% of patients experienced grade ≥ 3 TRAEs, with grade 3 QTc prolongation without ventricular arrhythmia in 8% and DS in 2%. These data highlight the profound responses to revumenib with a manageable safety profile [25].

### 4.2. Ziftomenib

Ziftomenib (KO-539) is another novel, once-daily, oral menin inhibitor targeting the menin–KMT2A protein–protein interaction. Interestingly, ziftomenib is metabolized to at least two metabolites with comparable activity to ziftomenib itself. The **KOMET-001** trial (**NCT04067366**) is an ongoing open-label phase 1/2A study evaluating ziftomenib monotherapy in adult patients with R/R acute leukemia [23]. No dose-limiting toxicities (DLTs) occurred during the dose-escalation phase of the study within the 28-day DLT assessment window. Grade 3 or higher TRAEs included grade 3 tumor lysis syndrome (TLS) at 50 mg and a grade 3 embolic event at 100 mg. Ziftomenib was well tolerated with no dose interruptions or discontinuations due to TRAEs. There were no treatment-related deaths [26,29].

A recent update of **KOMET-001** enrolled 30 adult patients with R/R AML with a median age of 65 years and a median of three prior therapies. Patients received various doses of ziftomenib to assess safety, tolerability, pharmacokinetics and antileukemic activity. The safety assessment shows comparable results to revumenib: TRAEs ≥ grade 3 were anemia (27%), pneumonia (27%), neutropenia (17%) and thrombocytopenia (13%), while two dose-limiting toxicities (DLTs) occurred: pneumonitis (400 mg) and DS (1000 mg) [26].

Of the 30 patients enrolled, 24 had KMT2Ar/NPM1mut AML. In this subset of patients, two doses (200 mg and 600 mg) were investigated to find the optimal biologically active dose. TRAEs ≥ grade 3 were observed in ≥10% of patients (N = 24) and included anemia, febrile neutropenia, neutropenia, thrombocytopenia, DS, leukocytosis, sepsis and leukopenia. TRAEs were similar in the 200 mg and 600 mg dose groups. The most common adverse event was DS, but prompt initiation of steroids and recognition of symptoms (unexplained fever, weight gain, edema, pleural or pericardial effusion, radiographic opacities, dyspnea, hypotension, renal dysfunction, rash, rapidly increasing white blood cell count) mitigated the effects of DS. Clinical efficacy was shown to be dose-dependent: at 600 mg, 25% of patients had the best response of CR or CR with partial hematologic recovery (CRh); 33.3% of NPM1mut patients achieved CR/CRh. Composite CR (CR + CRh + CRp) occurred in 33%, while the minimal residual disease negativity rate was 75% in patients with composite CR. The ORR was 42%, but patients who experienced DS showed an ORR of 75% [27].

The phase 1 **KOMET-001** trial showed a manageable safety profile for ziftomenib. Therefore, the 600 mg dose demonstrates meaningful efficacy in heavily pretreated R/R AML [27,28,29].

### 4.3. Other Menin Inhibitors under Investigation

Other menin inhibitors under evaluation in phase 1 clinical trials are **JNJ-75276617**, **BMF-219, DS1594 and DSP 5336** (Table 1). The last two drugs are in a very preliminary clinical phase [30,31].

The menin inhibitor JNJ-75276617 shows potent antiproliferative activity against a range of AML cell lines and patient samples harboring KMT2Ar or NPM1mut AML in vitro, with >95.5-fold selectivity over wild-type KMT2A/NPM1 AML [32]. Recently, some preliminary results were presented from a phase 1 study (**NCT04811560**) in adults with R/R KMT2Ar and NPM1mut acute leukemia. As of April 2023, fifty-eight patients had received JNJ-75276617 with encouraging preliminary results showing an acceptable safety profile and antileukemic activity as monotherapy, consistent with other menin inhibitors mentioned previously [33].

The menin inhibitor BMF-219 was tested in the phase I study **COVALENT-101 (NCT05153330)**, in R/R ALL and AML. At the data cut-off date of July 2023, 26 patients with R/R AL (24 AML; 2 ALL) had been enrolled. BMF-219 was well tolerated with no dose-limiting toxicities observed and no discontinuations due to treatment-related toxicities [34].

### 4.4. Menin Inhibitors and Venetoclax Combinations

The investigation of mechanisms of resistance to venetoclax-based therapies in AML, which is the current standard of care for elderly patients or those unable to receive intensive chemotherapy, showed an important activation of a KMT2A-like signature. These mechanisms of resistance are mediated by the upregulation of HOX and MEIS1 [35,36,37].

Therefore, menin inhibition downregulation of HOX and MEIS1 genes may lead to resistance to venetoclax being overcome in AML [5,35]. These findings pave the way for combinations of menin inhibitors and venetoclax.

Rausch et al. recently screened for synergistic treatment partners for ziftomenib, evaluating the combination of ziftomenib with other drugs (37 different compounds) to assess the synergistic leukemic cell killing of ziftomenib on KMT2Ar and NPM1mut AML. The results show synergistic effects of ziftomenib with many of the pre-selected drugs, particularly agents targeting epigenetic regulation and DNA damage, apoptosis and cell cycle (e.g., BCL, AKT and CDK4/6) and PARP inhibitors (olaparib, talazoparib). All-trans-retinoic acid (ATRA) was also highly synergistic in both AML subtypes. Among various combinations, ziftomenib plus venetoclax appeared to be the most interesting synergistic combination due to the additional downregulation of BCL2 with ziftomenib. Data from this study suggest that ziftomenib may induce apoptotic priming and resensitize AML cells to venetoclax. This finding may support further evaluation as a potential treatment option for AML patients with KMT2Ar or NPM1mut AML who have failed venetoclax-based regimens and have few other treatment options [10,35,36].

Interestingly, the phase 1 clinical trial **KOMET-007 (NCT05735184)** is currently ongoing to evaluate the safety, tolerability and preliminary antileukemic activity of ziftomenib in combination with venetoclax/azacitidine or standard induction chemotherapy (3 + 7) in newly diagnosed or R/R patients with NPM1mut and KMT2Ar AML. Initial results indicate the acceptable safety and efficacy of this combination in R/R myeloid leukemia with KMT2Ar or NPM1mut or NUP98r. This study is ongoing with plans to establish the phase 2 dose and optimize the administration of this combination [37].

Finally, Issa et al. reported early results from the **SAVE study (NCT05360160),** evaluating the combination of revumenib with decitabine/cedazuridine (ASTX727) and venetoclax in R/R AML or mixed-lineage acute leukemia (MPAL). Preliminary results show an acceptable safety profile and high efficacy of this combination of agents, with seven out of eight patients achieving morphologic remission and three out of seven patients achieving minimal residual disease negativity [38].

### 4.5. Menin Inhibitors and Other Combinations

In addition, another open-label dose-escalation and expansion study, KOMET-008, is planned to evaluate the safety, tolerability and preliminary efficacy of ziftomenib in combination with standard of care regimens for the treatment of NPM1mut or KMT2Ar R/R AML (FLAG-IDA, LDAC and gilteritinib in NPM1mut R/R AML; FLAG-IDA and LDAC in KMT2Ar R/R AML) [39]. 

There is also a rationale for combining FLT3 inhibitors and menin inhibitors based on the downregulation of FLT3 transcription in patients with NPM1mut AML treated with menin inhibitors [40]. FLT3 downregulation after the inhibition of menin–KMT2Ar binding is associated with a dramatic reduction in MEIS1 expression. Therefore, the pronounced reduction in FLT3 after the combination of menin–KMT2Ar binding inhibition and FLT3 inhibition resulted in the significantly enhanced suppression of STAT5A target genes. STAT5A contributes to leukemia maintenance and is an important downstream mediator of activating FLT3 mutations [40].

## 5. Conclusions

Menin inhibitors represent an exciting new class of agents against KMT2Ar and NPM1mut leukemias. Early data from ongoing clinical trials show promising results in terms of response rates and safety in heavily pretreated patients. However, further studies are needed to confirm the efficacy data, to identify subgroups of patients in whom these agents may be more effective, and to determine the optimal timing of administration and doses of these new drugs.

In addition, menin inhibitors appear to have a synergistic effect with many other agents, such as venetoclax and FLT3 inhibitors, enhancing the effects of both classes of drugs and potentially improving outcomes in KMT2Ar and NPM1-mutated AML, especially in older patients and in those with R/R AML. In summary, we have new promising players in the fight against acute leukemia.

## Figures and Tables

**Table 1:** Menin inhibitors currently available in phase I/II clinical trials for patients with acute leukemia.

Drug	Trial (ClinicalTrials.gov ID)	Phase	Regimen	Patient Population	Eligibility
**Revumenib** **(SNDX-5613)**	**AUGMENT-101** **(NCT04065399)**	I/II	Revumenib monotherapy	131 Adults and children	R/R KMT2Ar AL, NPM1c AML
	AUGMENT-102 (NCT05326516)	I	-AML: Revumenib/FLA ± Revumenib/FLA -ALL/MPAL: Revumenib/Pred/VCR/ASP/DNR	30 Adults and children	R/R KMT2Ar AL, NPM1c or NUP98r AML
	(NCT05761171)	II	Revumenib/FLA, MTX	Children (recruiting)	R/R KMT2Ar ALL
	**SAVE ** **(NCT05360160)**	I/II	Revumenib/ASTX727/VEN	8 Adults and children (recruiting)	R/R AML or MPAL
	BeatAML substudy (NCT03013998)	I	Revumenib/VEN/AZA	13 Adults	Newly diagnosed KMT2Ar or NPM1c AML
**Ziftomenib** **(KO-539)**	**KOMET-001** **(NCT 04067336)**	I/II	Ziftomenib monotherapy	30 Adults	Phase 1a: R/R AML Phase 1b/2: KMT2Ar or NPM1c AML
	**KOMET-007** **(NCT05735184)**	I	-Newly diagnosed AML: Ziftomenib/7 + 3 -R/R AML: ziftomenib/VEN/AZA	20 Adults	Newly diagnosed and R/R KMT2Ar or NPM1c AML
**JNJ-75276617**	**NCT04811560**	I	JNJ-75276617 monotherapy	58 Adults	R/R KMT2Ar AL, NPM1c AML
	NCT05453903	I	JNJ-75276617/VEN, JNJ-75276617/AZA or JNJ-75276617/VEN/AZA	Adults (recruiting)	R/R KMT2Ar AL, NPM1c AML
	NCT05521087	I	AML: JNJ-75276617/FLA ALL: JNJ-75276617/DEX/VCR/ASP	Adults and children (recruiting)	R/R KMT2Ar AL, NPM1c ALL/AML, NPM1c or NUP98r AML
**BMF-219**	**COVALENT-101** **(NCT05153330)**	I	BMF-219 monotherapy	26 Adults	R/R AL, DLBCL or MM
**DS-1594**	NCT04752163	I/II	DS-1594 = AZA, VEN or mini-HCVD	17 Adults	Phase 1: R/R AL Phase 2: R/R KMT2Ar ALL/AML, NPM1c AML
**DSP-5336**	NCT04988555	I/II	DSP-5336 monotherapy	4 Adults	Phase 1: R/R AL Phase 2: R/R KMT2Ar ALL/AML, NPM1c AML

From clinicaltrials.gov. AL, acute leukemia; ALL, acute lymphoblastic leukemia; AML, acute myeloid leukemia; ASP, PEG-asparaginase; AZA, azacytidine; CPM, cyclophosphamide; DEX, dexamethasone; DNR, daunorubicine; DLBCL, diffuse large B-cell lymphoma; ETO, etoposide; FLA, fludarabine and cytarabine; MPAL, mixed-phenotype acute leukemia; mini-HCVD, cyclophosphamide, cytarabine, dexamethasone, methotrexate, prednisolone, rituximab and vincristine; MTX, methotrexate; MM, multiple myeloma; Pred, prednisolone; R/R, refractory or relapse; VCR, vincristine; VEN, venetoclax; 7 + 3, cytarabine and anthracycline.

## Data Availability

Not applicable.
